# Detection of fatty liver using virtual non-contrast dual-energy CT

**DOI:** 10.1007/s00261-022-03482-9

**Published:** 2022-03-19

**Authors:** Pengcheng Peter Zhang, Hailey H. Choi, Michael A. Ohliger

**Affiliations:** 1 Department of Radiology and Biomedical Imaging, University of California, San Francisco, 505 Parnassus Avenue, 3rd Floor, M372, San Francisco, CA 94143 USA; 2 Department of Radiology, Zuckerberg San Francisco General Hospital, 1001 Potrero Ave, SFGH 5 Room 1x56, Box 0628, San Francisco, CA 94110 USA

**Keywords:** Dual energy, NAFLD, NASH, Fatty liver, PDFF, Liver

## Abstract

**Purpose:**

Determine whether liver attenuation measured on dual-energy CT (DECT) virtual non-contrast examinations predicts the presence of fatty liver.

**Methods:**

Single-institution retrospective review from 2016 to 2020 found patients with DECT and proton density fat fraction MRI (MRI PDFF) within 30 days. MRI PDFF was the reference standard for determining hepatic steatosis. Attenuation measurements from VNC and mixed 120 kVp-like images were compared to MRI PDFF in the right and left lobes. Performance of VNC was compared to measurement of the liver-spleen attenuation difference (LSAD).

**Results:**

128 patients were included (69 men, 59 women) with mean age 51.6 years (range 14–98 years). > 90% of patients received CT and MRI in the emergency department or as inpatients. Median interval between DECT and MRI PDFF was 2 days (range 0–28 days). Prevalence of fatty liver using the reference standard (MRI PDFF > 6%) was 24%. Pearson correlation coefficient between VNC and MRI- DFF was -0.64 (right) and -0.68 (left, both *p* < 0.0001). For LSAD, correlation was − 0.43 in both lobes (*p* < 0.0001). Considering MRI PDFF > 6% as diagnostic of steatosis, area under the receiver operator characteristic curve (AUC) was 0.834 and 0.872 in the right and left hepatic lobes, with an optimal threshold of 54.8 HU (right) and 52.5 HU (left), yielding sensitivity/specificity of 57%/93.9% (right) and 67.9%/90% (left). For LSAD, AUC was 0.808 (right) and 0.767 (left) with optimal sensitivity/specificity of 93.3%/57.1% (right) and 78.6%/68% (left).

**Conclusion:**

Attenuation measured at VNC CT was moderately correlated with liver fat content and had > 90% specificity for diagnosis of fatty liver.

**Graphical abstract:**

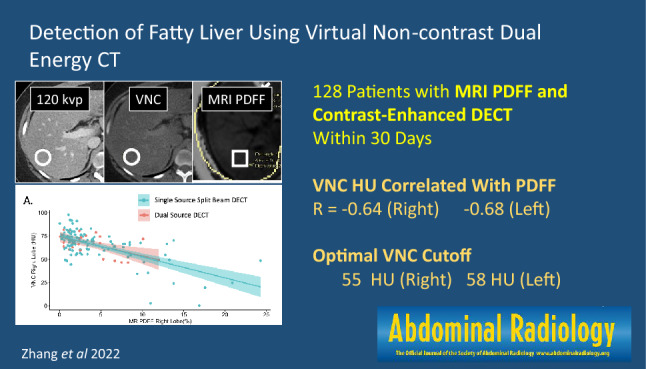

## Introduction

Fatty liver disease is characterized by accumulation of lipid in the liver and affects 30–40% of the US population [1]. This disorder is associated with obesity and type 2 diabetes mellitus [1, 2]. Without dietary modification or other therapeutic interventions, fatty liver disease can progress to steatohepatitis and cirrhosis. Even in its non-progressive form, fatty liver disease is associated with increased all-cause mortality [3]. Fatty liver disease and non-alcoholic steatohepatitis (NASH) are currently the second most common indication for liver transplantation [4]. Due to the increasing prevalence of obesity and the decline in hepatitis C with curative therapies, fatty liver disease and NASH are expected to become the most common indications for liver transplantation [1, 2].

Traditionally, liver biopsy has been the gold standard for the diagnosis and quantification of hepatic steatosis. More recently, MRI-based methods of fat quantification have emerged as non-invasive and reliable alternatives to biopsy. MRI proton density fat fraction (PDFF) is a technique for chemical shift-based water and fat separation [5], correcting for the effects of magnetic susceptibility (i.e., iron deposition), magnetic field inhomogeneity, and multiple different lipid peaks that occur in liver [6]. MRI PDFF is highly correlated with liver biopsy and histology [7]. Additionally, MRI PDFF could reduce sampling error and intra-observer error related to liver biopsy.

Although MRI is an excellent quantitative tool for following patients with known fatty liver disease, it is limited as a screening tool due to expense and availability. By contrast, computed tomography (CT) is widely available, and many patients will undergo CT for indications unrelated to liver disease, such as the evaluation of abdominal pain in the emergency department. Therefore, quantitative and reliable CT-based techniques have the potential to have broad impact on patient care. When performed without intravenous contrast, CT attenuation < 48 HU is specific for mild and moderate steatosis [8–10]. However, contrast-enhanced CT (CECT) is more commonly performed in routine practice for a variety of clinical indications. The presence of contrast within the liver and differences in contrast bolus timing alters hepatic attenuation and reduces the specificity of contrast-enhanced CT for hepatic steatosis [6, 11]. Although a liver-spleen attenuation difference on CECT greater than 20 HU is specific for moderate steatosis (> 30%), specificity is much lower for mild steatosis [6, 8, 10, 12, 13]. Additionally, the presence of other materials in the liver, such as iron, may also lead to underestimation of hepatic steatosis [5].

DECT relies on attenuation measurements of x-rays at multiple different energies. Different materials and tissues have unique characteristic attenuation profiles, especially for higher-energy x-rays [5]. Differences in material-specific attenuation at each energy level allow material decomposition algorithms to produce data on specific materials present, such as fat and iodine. Additionally, DECT with subtraction of iodine from post-contrast images permits generation of virtual non-contrast (VNC) images, allowing for measurement of attenuation for evaluation of hepatic steatosis.

Multiple methods are available for acquiring dual-energy CT: (1) Dual-source DECT uses two x-ray tubes and two sets of detectors; (2) Single-source DECT with rapid kilovoltage switching uses a single x-ray tube that rapidly alternates between energies; (3) Split-beam DECT uses two filters to the x-ray beam at its source; and (4) Detector-based DECT has two layers of detectors that collect low- versus high-energy photons [14].

DECT has been tested using phantoms with known fat content and in animals with histologic correlation [14–16]. A recent retrospective study in human subjects compared DECT to subjective grading of steatosis as mild, moderate, or severe based on conventional ultrasound or CT [17]. VNC images were shown to match true non-contrast images in both abdominal phantoms [18] as well as the livers of patients undergoing planning for transcatheter aortic valve replacement [19]. Other retrospective studies have investigated DECT for fatty liver without a reference standard, i.e., no quantitative MRI or liver biopsy for comparison [20–22]. DECT without intravenous contrast has been compared prospectively to MRI PDFF and MRS in a large cohort of patients receiving CT colonography [23]; however, the performance of contrast-enhanced DECT was not measured [23]. Another study correlated DECT to MRI PDFF in a small (19-patient) cohort of NAFLD patients [23, 24]. However, since the cohort already had a NAFLD diagnosis, prospective performance in diagnosing fatty liver was not tested.

The goal of our study was to determine if DECT post-processed virtual non-contrast imaging could accurately determine hepatic steatosis, using MRI PDFF as reference.

## Materials and methods

### Population

This retrospective study was performed with approval of the local institutional review board. Written informed consent was waived. Single-center, retrospective review of the PACS database revealed 205 pairs of DECT and MRI performed within 30 days of each other between 2016 and 2020. Examinations were excluded if MR images had “fat water swap” artifacts [25]. When multiple CT examinations were performed within 30 days of MR (or vice versa) for the same patient, the DECT and MRI examinations performed within the shortest time interval were used for analysis.

### Imaging technique

CT imaging was performed as part of routine clinical care on one of three different DECT scanners: two scanners used were split-beam DECT scanners (Somatom Definition Edge, Siemens Healthineers, Au 120 KvP/ Sn 120 KvP) and one was a 2nd generation dual-source CT scanner (Somatom Definition Flash, Siemens Healthineers, 100 KvP/140 KvP). All images were obtained according to the institution’s standard abdominal imaging protocol, following intravenous administration of 150 mL iohexol (350 mg iodine/mL) in the portal venous phase, approximately 80 s after contrast administration. Contrast rate varied depending on the IV access available and ranged from 1 to 3 mL/s. For the split-beam DECT scanners, pitch was 0.45 with rotation time 0.28 s. For the dual-source DECT scanner, pitch was 1.2 and rotation time was 0.28 s.

MR PDFF scans were acquired as part of routine clinical care at 3 T using a multi-echo gradient echo pulse sequence (LiverLab, Siemens Healthineers) with 6 echoes. Sequence parameters were as follows: repetition time 9 ms, flip angle 4°, field of view 38 cm, matrix size 111 × 160, and echo times 1.09 ms, 2.46 ms, 3.69 ms, 4.92 ms, 6.15 ms, and 7.38 ms.

VNC and iodine maps were generated using vendor-supplied software (Siemens Syngovia) using the “Liver VNC” setting. Regions of interest (ROI) were placed in the right and left hepatic lobes of the “mixed” (120 kVp-equivalent) images. Each ROI had an area measuring approximately 5–6 cm^2^. ROIs were placed in central regions of the right and left hepatic lobes, and care was taken to avoid placing ROIs on hepatic vasculature. For each ROI, the HU of the VNC image as well as the mixed 120 kVp-equivalent image were recorded. ROIs were subsequently placed in matching locations within the right and left hepatic lobes within MRI-derived fat fraction maps. Examples of ROI placement in situations of normal and elevated liver fat are illustrated in Fig. 2.

In order to compare VNC-derived measurements with conventional CT measurements (120 kVp-like combined images), liver-spleen attenuation differences (LSAD) were determined using ROIs from the right and left hepatic lobes as well as an additional ROI that was placed over the spleen.

The indications for MRI examinations were reviewed by a single abdominal radiologist with 9 years of experience and categorized as follows: (1) Cholecystitis (known or suspected), (2) Gallbladder or common bile duct stones (known or suspected), (3) Dilated common bile duct, (4) Fever and cholangitis (known or suspected), (5) Biliary obstruction suspected, (6) Focal mass, (7) Pain, (8) Abscess, (9) Abnormal liver function tests, (10) Pancreatitis (known or suspected), and (11) Other. All applicable categories were chosen, meaning a given study could have more than one category assigned. Categories were tabulated and reported as a percentage of total studies.

### Analysis and statistics

Statistical analysis was performed using the programming language R (R Core Team, 2017; Vienna, Austria, www.R-project.org). The right and left hepatic lobe measurements were analyzed separately. Data were analyzed for linear correlation between VNC HU and MRI PDFF using the Pearson correlation coefficient. Degree of correlation was defined based on the correlation coefficient as follows: Very strong 0.9–1.0, Strong 0.7–0.89, Moderate 0.4–0.69, Weak 0.1–0.39, and Negligible 0.0–0.09 [26]. Receiver operator characteristic (ROC) analysis was also performed separately for right and left lobes. Considering 6% as the upper limit of normal fat fraction on MRI PDFF, the optimal threshold (Youden’s index) for VNC HU was calculated for the right and left lobes, respectively. 95% confidence intervals were computed for the area under the ROC curves using the DeLong method. In order to compare DECT-based measurements with conventional measurements, an identical analysis was performed using the LSAD. Test characteristics (sensitivity, specificity) for diagnosing hepatic steatosis were also determined using the optimal VNC HU cut-off values as determined by ROC analysis, as well as the commonly used cut-off for LSAD of greater than − 20 HU [6, 8, 10, 12, 13]. Finally, a subgroup analysis was performed by individually evaluating examinations of each scanner type (split-beam DECT and dual-source DECT) with regard to our primary research question, which was the correlation of VNC values to MR PDFF values.

## Results

**Fig. 1:**
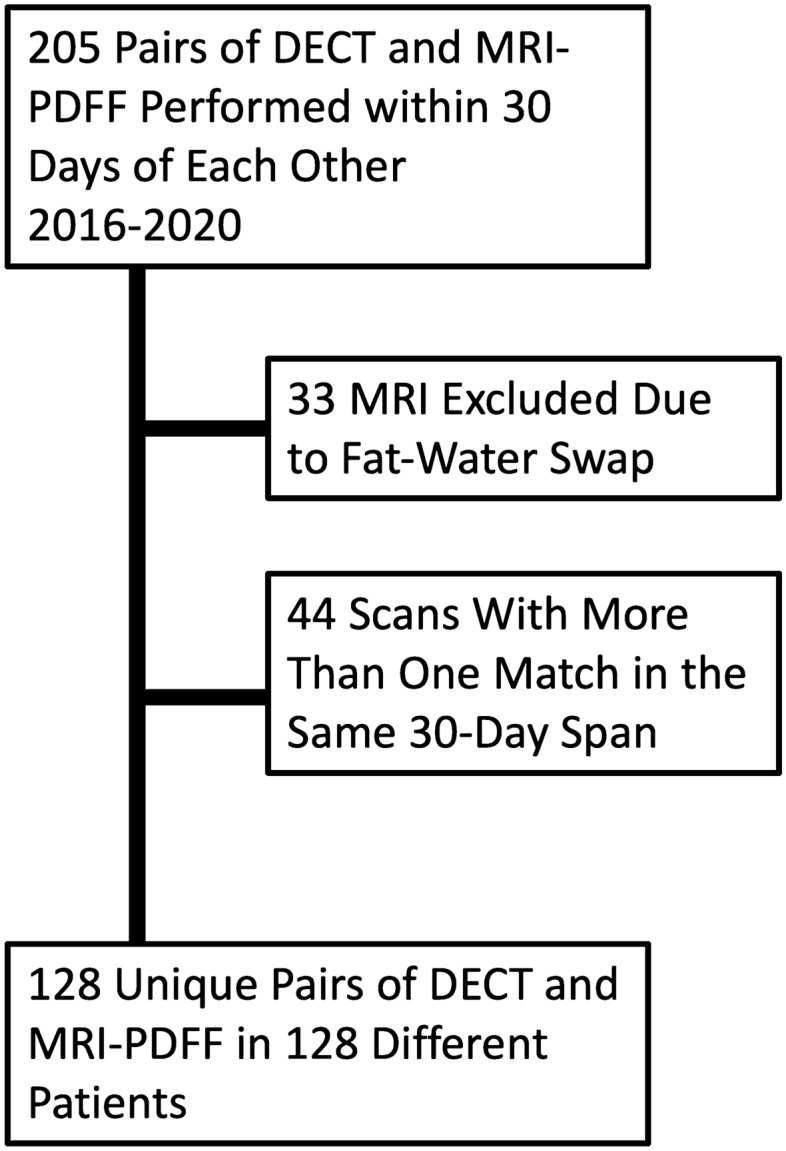
Flow diagram with study inclusion and exclusion criteria. *DECT* dual-energy CT, *PDFF* proton density fat fraction

**Fig. 2:**
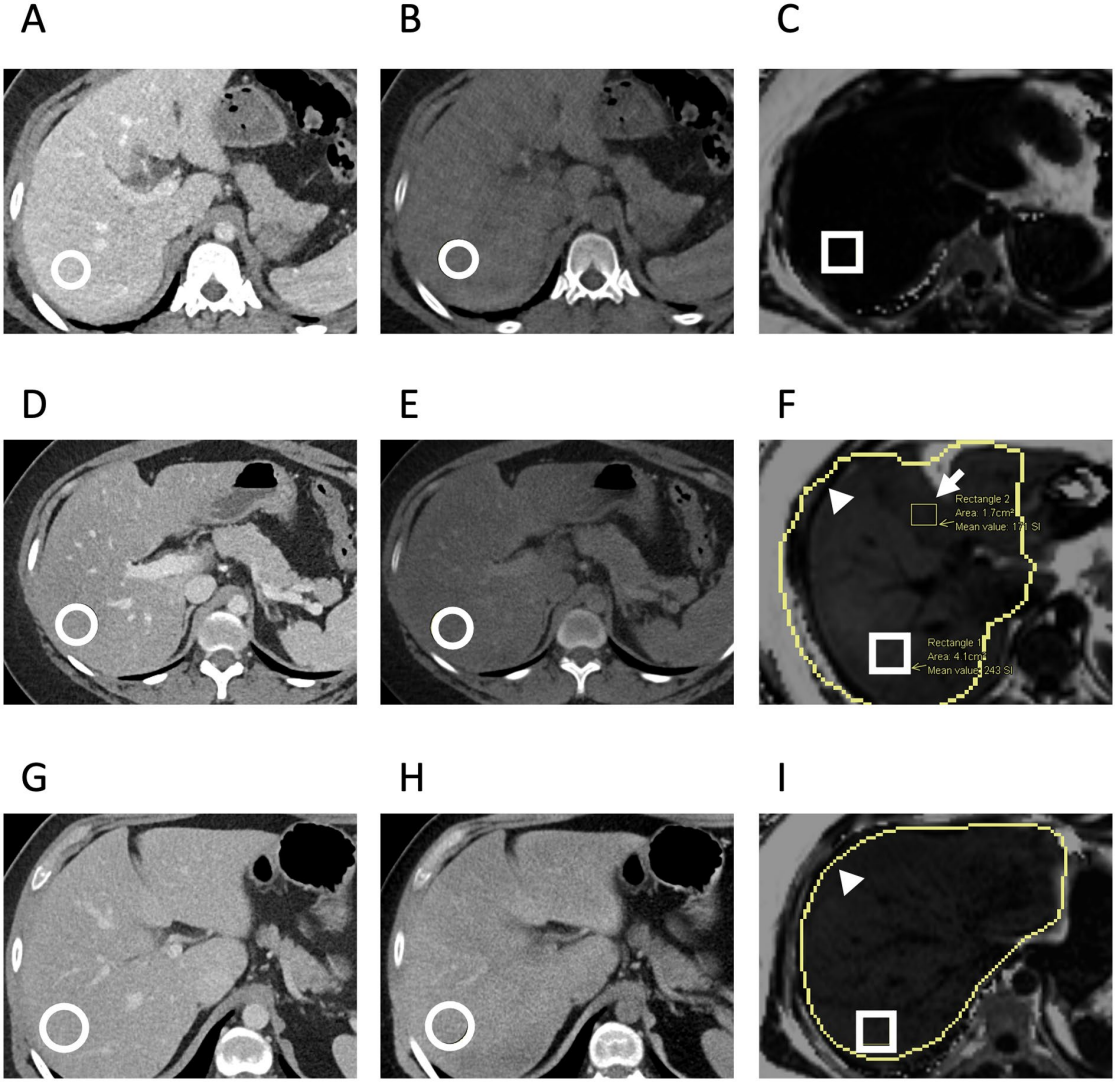
Representative patient images used for analysis. **a** Concordant-negative example: single axial slice from the combined 120 kVp-equivalent 30-year-old inpatient using dual-source DECT. **b** VNC DECT image with a circular ROI placed in the right hepatic lobe (82 HU). **c** MRI PDFF image with an ROI in the same location shows no fatty liver (PDFF = 0.5%). **d** Concordant-positive example: 120 kVp-equivalent axial image from a 29-year-old scanned in the emergency department using a split-beam DECT. **e** DECT VNC image with ROI placed in the right hepatic lobe showing decreased attenuation (24.3 HU). **f** MRI PDFF image at the same location showing markedly elevated liver fat (PDFF = 24.3%). This figure also contains the ROI used for the left lobe in this subject (arrow). The anatomically matched corresponding CT ROI was located on a different slice. **g** Discordant example: 120 kVp-equivalent axial image from a 30-year-old scanned in the emergency department using a split-beam DECT. **h** DECT VNC image with ROI placed in the right hepatic lobe showing normal attenuation (70.4HU). **i** MRI PDFF image at the same location showing elevated liver fat (PDFF = 13.8%). The outer yellow contours in panels **f** and **i** (arrowheads) correspond to the vendor’s automated liver segmentation (burned into the image) and was not part of our analysis. *DECT* dual-energy CT, *VNC* virtual non-contrast, *PDFF* proton density fat fraction

205 pairs of DECT and MRI PDFF performed within 30 days of each other occurred at our institution between 2016 and 2020. 33 pairs were excluded due to the presence of “fat–water swap” artifact in MR images [25]. In 44 cases, more than one pair of DECT and MRI were performed within a 30-day interval for the same patient; for these, the two closest exams were chosen (Fig. 1). The final study population included 128 unique pairs of CT and MRI examinations occurring in 128 patients (each patient had only one qualifying pair of examinations), with patient characteristics summarized in Table 1. 69/128 (54%) of patients were female. Mean age was 52 years (standard deviation 17 years, range 14–89 years). Nearly all patients’ CT examinations were performed in the emergency department (80%) or as inpatients (18%). In terms of the type of DECT scanner used, 102/128 (80%) of patients received “split beam” DECT, and 26/128 (20%) of patients received dual-source DECT. The vast majority of MRI examinations (90%) were performed in the inpatient setting (Fig. 2). The median time between CT and MRI examination was 2 days (range 0–28). The most common indication for MRI was pain (34/128, 26.5%), followed by known or suspected gallbladder/common bile duct stones (27/128, 21.1%) and abnormal liver function tests (24/128, 18.8%) (Table 2).

**Table 1:** Patient demographics and setting of imaging

Total patients	128
Female	69
Male	59
Mean age (range)	51.6 (14, 89)
Median time interval between DECT and MRI PDFF (days) (range)	2 (0, 28)
*Clinical context of DECT*	
Emergency department	102
Inpatient	23
Outpatient	3
*Clinical context of MRI PDFF*	
Inpatient	115
Outpatient	13

*DECT* dual-energy CT, *PDFF* proton density fat fraction

**Table 2:** Indications for MRI examinations

Category	Number	Percent of total (%)
Cholecystitis (known or suspected)	8	6.3
Gallbladder or common bile duct stones (known or suspected)	27	21.1
Dilated common bile duct	15	11.8
Fever and cholangitis (known or suspected)	5	3.9
Biliary obstruction suspected	10	7.8
Focal mass	18	14.1
Pain	34	26.5
Abscess	4	3.1
Abnormal liver function tests	24	18.8
Pancreatitis	14	14.1
Other	15	11.7

Note that because some exams fit more than one category, the total is greater than 100%

**Fig. 3:**
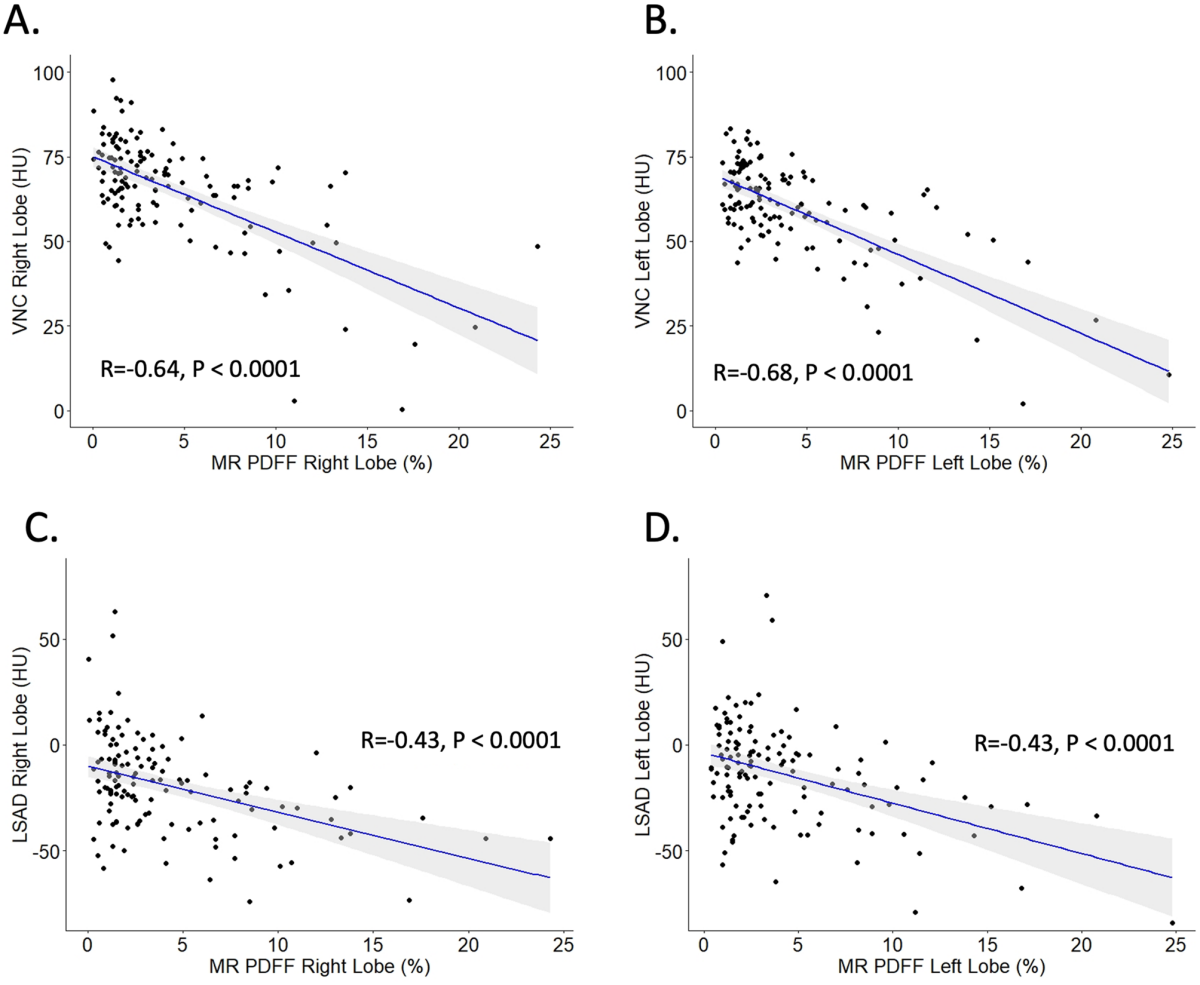
**a** Scatterplots demonstrating correlation between VNC HU and MRI PDFF in the right and **b** left lobes of the liver. **c** Scatterplots showing correlation between LSAD and MRI PDFF in the right and **d** left lobes of the liver. Pearson correlation coefficients and *p* values are displayed. *VNC* virtual non-contrast, *PDFF* proton density fat fraction, *LSAD* liver-spleen attenuation difference

There was a moderate degree of negative correlation between VNC HU on DECT and PDFF on MRI, with Pearson correlation coefficients of − 0.64 (95% CI − 0.52, − 0.73) for the right lobe (*p* < 0.001, Fig. 3a) and − 0.68 (95% CI − 0.57, − 0.76) for the left lobe (*p* < 0.001, Fig. 3b). Correlation was lower, but still moderate, for the traditional method of LSAD, with coefficients of − 0.43 for both right and left lobes (*p* < 0.001, Fig. 3c and d) (Table 3).

**Table 3:** Pearson correlation coefficient comparing VNC HU to MRI PDFF, parenthesis indicates 95% CI

	VNC HU	*p* value
Right lobe		
DECT VNC HU dual source	− 0.58 (− 0.25, − 0.79)	< 0.01
DECT VNC HU split beam	− 0.64 (− 0.51, − 0.74)	< 0.001
DECT VNC HU all scanner types	− 0.64 (− 0.52, − 0.73)	< 0.001
LSAD	− 0.43 (− 0.27, − 0.56)	< 0.001
Left lobe		
DECT VNC HU dual source	− 0.43 (− 0.06, − 0.70)	0.027
DECT VNC HU split beam	− 0.70 (− 0.59, − 0.79)	< 0.001
DECT VNC HU all scanner types	− 0.68 (− 0.57, − 0.76)	< 0.001
LSAD	− 0.43 (− 0.28, − 0.56)	< 0.001

**Fig. 4:**
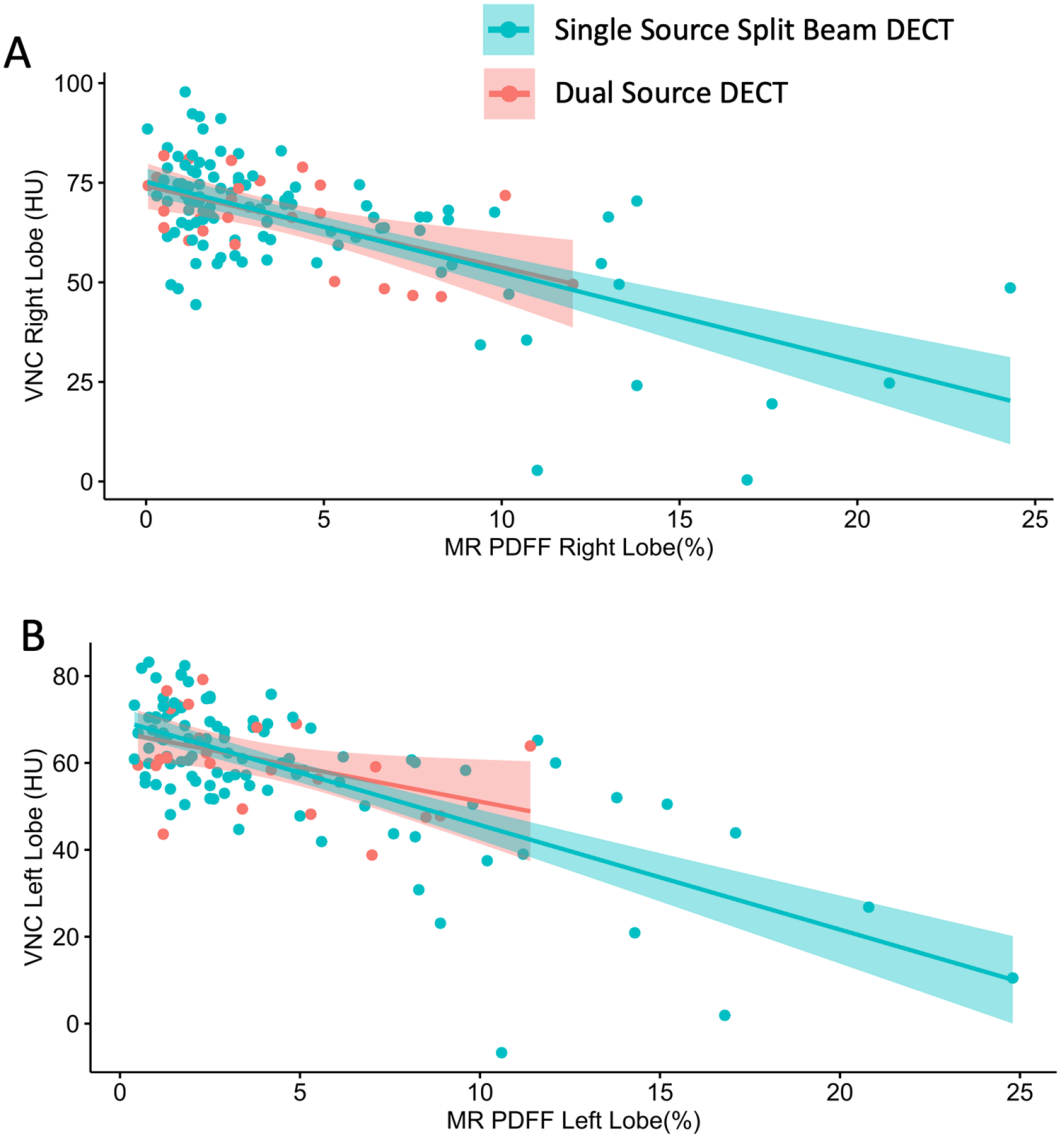
**a** Scatterplots demonstrating correlation between VNC HU and MRI PDFF in the right and **b** left lobes of the liver, broken down by scanner type. Correlation values as follows: See Table 3 for correlation values

In a subgroup analysis, CT scans performed with the single-source dual-energy scanners (split beam) had nearly identical correlation coefficients (− 0.64 in the right lobe, − 0.70 in the left lobe) to those of the full data set (Fig. 4a, Table 3). CT scans performed with the dual-source dual-energy scanner had larger differences in correlation coefficients from the full data set (− 0.58 in the right lobe, − 0.43 in the left lobe), but the 95% confidence intervals nearly completely overlapped (Fig. 4b, Table 3).

Considering MRI PDFF greater than 6% as indicative of liver steatosis, 30/128 (23%) of patients had steatosis in the right lobe and 28/128 (22%) of patients had steatosis in the left lobe. In the right lobe, average (± standard deviation) VNC HU was 50 HU (± 20 HU) for patients with steatosis and 70 HU (± 10) for patients without steatosis (*p* < 0.001). In the left lobe, average (± standard deviation) VNC HU was 43 HU (± 19 HU) for patients with steatosis and 64 HU (± 9 HU) for patients without steatosis (*p* < 0.001).

**Fig. 5:**
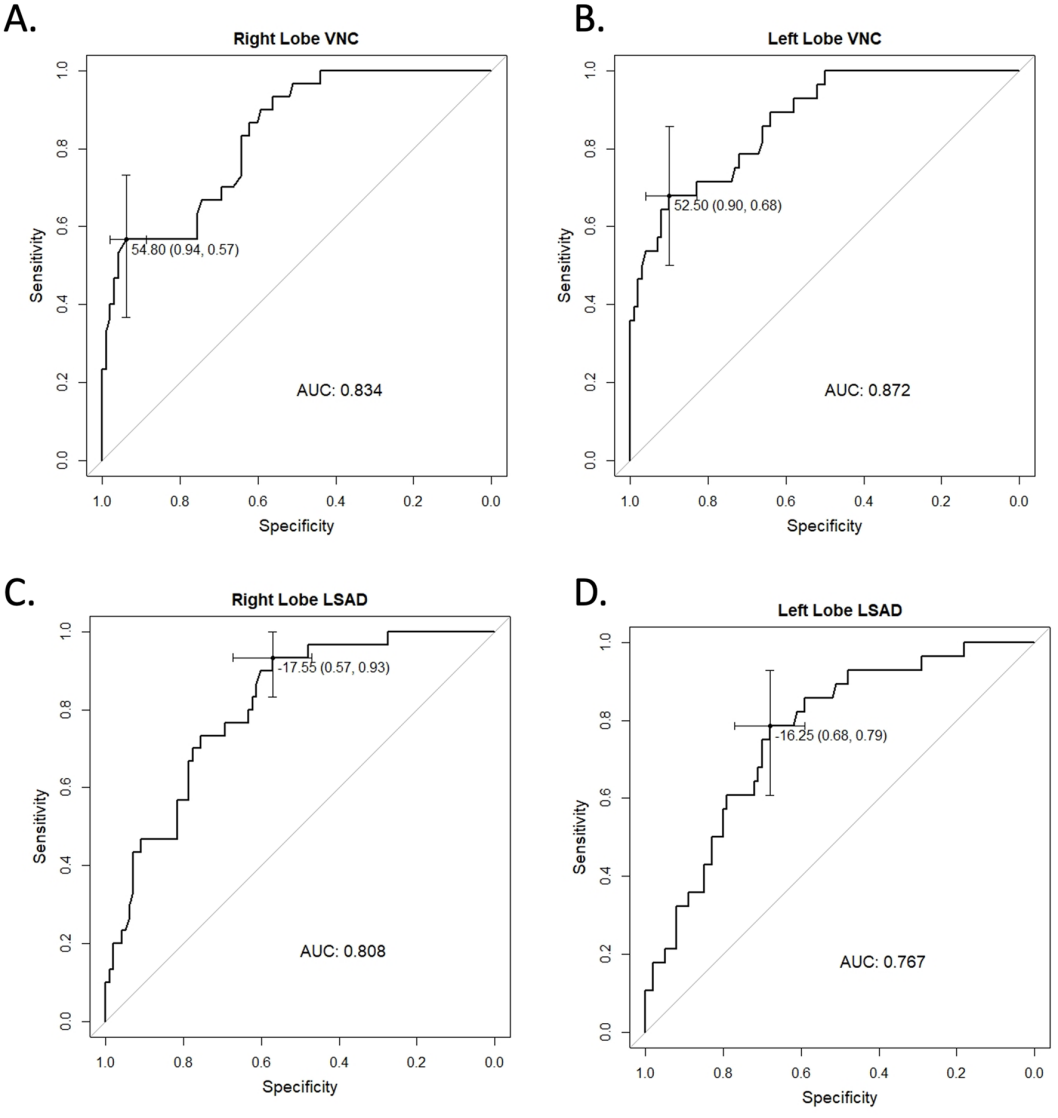
**a** Receiver operator characteristic (ROC) curve for VNC HU diagnosis of steatosis in the right hepatic lobe and **b** left hepatic lobe. **c** ROC curve for the diagnosis of steatosis using PDFF in the right hepatic and **d** left hepatic lobe. For all curves, MRI PDFF > 6% was used as the reference standard for diagnosis of steatosis. Areas under the curve (AUC) and optimal cut-off values with 95% confidence interval (whiskers) are displayed. The corresponding specificity and sensitivity are shown as coordinates for the optimal cut-off points

ROC analysis was performed for VNC HU, using MRI PDFF > 6% as the reference cut-off for establishing liver steatosis (Fig. 5a and b). For the right lobe, the area under the ROC curve (AUC) was 0.834 (95% CI 0.756–0.912), yielding an optimal threshold VNC HU cut-off of 54.8, with a specificity of 94% and sensitivity of 57%. For the left lobe, the AUC was 0.872 (95% CI 0.801–0.942). Optimal cut-off VNC HU was 52.5, resulting in a specificity of 90% and sensitivity of 68% (Table 4).

**Table 4:** Test characteristics of suggested VNC cu-toff vs liver-spleen difference

Right lobe	VNC HU threshold of 54.8	Liver-spleen difference less than-20 HU
Sensitivity	57% (37%, 75%)	87% (69%, 96%)
Specificity	94% (87%, 98%)	61% (51%, 71%)
Positive predictive value	74% (52%, 90%)	41% (29%, 54%)
Negative predictive value	88% (80%, 93%)	94% (85%, 98%)
ROC area under the curve	0.834 (0.756, 0.912)	0.808 (0.728, 0.889)

Left lobe	VNC HU threshold of 52.5	Liver-spleen difference less than-20 HU
Sensitivity	68% (48%, 84%)	68% (48%, 84%)
Specificity	90% (82%, 95%)	71% (61%, 80%)
Positive predictive value	66% (46%, 82%)	40% (26%, 55%)
Negative predictive value	91% (83%, 96%)	94% (80%, 95%)
ROC area under the curve	0.872 (0.801, 0.942)	0.767 (0.674, 0.861)

95% Confidence interval in parenthesis.

*VNC* virtual non-contrast, *ROC* receiver operator characteristic.

In comparison, the conventional measurement of LSAD on contrast-enhanced images revealed an AUC of 0.808 (95% CI 0.728–0.889) for the right lobe (Fig. 5c) and 0.768 (95% CI 0.674–0.861) for the left lobe (Fig. 5d). Optimal cut-off value for LSAD was − 17.6 in the right lobe and − 16.3 in the left lobe, yielding sensitivities of 93% (right) and 79% (left) and specificities of 57% (right) and 68% (left). When the commonly used LSAD cut-off of -20 HU was considered, sensitivity was 87% in the right lobe and 68% in the left. Specificity in the right and left lobe were 61% and 71%, respectively (Table 4).

## Discussion

In our study we found moderate correlation (Pearson coefficient 0.64–0.68) between DECT VNC and fat fraction measured by MRI in both the right and left hepatic lobes. When MRI PDFF > 6% was used as a threshold for diagnosing steatosis, we determined optimal VNC cut-off values of 54.8 HU for right lobe and 52.5 HU for left lobe, both of which showed high (> 90%) specificity for the diagnosis of hepatic steatosis. LSAD measured using conventional contrast-enhanced CT measures was more sensitive than DECT VNC but less specific.

In current practice, fatty liver disease is a clinical diagnosis based on laboratory markers, clinical history, radiologic, and pathologic evaluation [27]. Imaging-related diagnosis can be made with ultrasound, CT, and MRI. MRI with spectroscopy and MRI PDFF are sensitive and specific for the evaluation of hepatic steatosis. However, many patients with fatty liver disease are asymptomatic, and those without known risk factors may not undergo early screening with US or MRI. This may result in delayed or missed diagnosis of a potentially reversible cause of chronic liver disease. CT (in particular, contrast-enhanced CT) is often performed in the inpatient and emergent settings for evaluation of abdominal pain or other symptoms. While prior studies have shown limited sensitivity and specificity of contrast-enhanced CT for detection of fatty liver, DECT and material decomposition may improve early incidental detection. Given the high prevalence of fatty liver disease, involving 30% of the US population [28], early incidental detection could direct patients to appropriate medical care and reduce progression to steatohepatitis and cirrhosis.

Previous studies using phantoms and animal models also showed correlation between DECT VNC attenuation and hepatic steatosis [5, 15, 16, 29, 30]. Prior studies investigating DECT evaluation of liver fat in humans have used varying reference standards and patient populations [17, 20–22, 24]. Some studies used true non-contrast CT images, while others used ultrasound, LSAD, and MRI PDFF. One prospective study investigating hepatic steatosis was performed using DECT and MRS performed on the same day [23]. However, DECT was performed without intravenous contrast. A second retrospective study, performed using patients with a known diagnosis of NAFLD with CT and MRI within 3 days of each other, found a high correlation between DECT-derived fat content and MRI PDFF [24]. However, the number of patients [21] was smaller than the current study; and because patients all had a known diagnosis of NAFLD, the performance in detecting the presence of fatty liver disease could not be assessed.

Advantages of our study include a moderately large sample size (*n* = 128), as well as a short median interval of 2 days between DECT and MRI PDFF. Additionally, the prevalence of fatty liver in our study population (24%) was similar to the general population [28]. We employed three different CT scanners using two different DECT technologies. We used MRI PDFF as reference, which is a widely accepted technique that has been validated against MR spectroscopy and biopsy. We also compared the diagnostic performance of DECT to LSAD—a commonly used, conventional method for detecting fatty liver on standard contrast-enhanced images. Of note, the optimal cut-off for LSAD derived from our study on conventional contrast-enhanced images (− 17.6 HU and − 16.3 HU in the right and left lobes, respectively), matched the commonly used threshold of − 20 HU that is commonly used in current clinical practice, further confirming the representative nature of our study samples.

The use of two different types of DECT scanners (split beam and dual source) has the potential to confound results if the two types of scanners have drastically different performance. Indeed, there is evidence that the split-beam DECT and dual-source DECT may give different HUs in the liver [31]. At the same time, the use of multiple scanner types is reflective of a real-world situation where different scanners may be used in the same institution. A subgroup analysis performed on data from each type of scanner showed nearly identical correlation when examining the full data set and when restricting the analysis to scans from the single-source split-beam scanner. There were not enough scans using the dual-source DECT scanners to directly compare the performance of the two scanner types, although the correlation coefficients derived from the two different scanners had overlapping confidence intervals. Scanner-specific diagnostic performance may be an important topic for future studies.

While liver biopsy is the gold standard for evaluation of fatty liver disease, some patients may not be able to undergo this invasive evaluation due to associated risks, such as bleeding, bile leak, and anatomic limitations.

DECT VNC and MRI PDFF measurements were made in the central liver, with one ROI placed in the right lobe and one ROI placed in the left lobe. We examined the two lobes separately in order to allow for the possibility that a different correlation could be present in each lobe. Some areas of the liver such as the lateral segment of the left hepatic lobe may conceivably be more susceptible to cardiac motion and therefore give less reliable results, but this was not systematically studied. This is a potential topic for future studies.

MRI PDFF was measured in two selected ROIs that were chosen to match the ROIs used for CT. Although larger ROIs that encompass the whole liver are often used for the diagnosis of fatty liver disease, our priority was correlating the VNC HU in a specific location with MRI PDFF measured in that location. It has been shown that a small number of ROIs chosen in a “balanced” fashion in the right and left lobes can be representative of the liver fat fractions measured over all segments, although four ROIs would potentially be more reliable than two [32]. This is a potential topic for future studies.

Although non-enhanced CT is known to be reliable for detection of moderate steatosis, detection on contrast-enhanced studies is limited due to variations in contrast bolus and scan timing, cardiac output, and other factors [6, 8–11]. A specific examination and threshold HU could help to identify hepatic steatosis earlier and direct patients to appropriate care. Although further processing of the DECT images to generate “fat maps” has been described and are available as clinical products, we focused on VNC attenuation values because of their conceptual similarities to currently accepted methodologies using true non-contrast examinations [21, 22, 24]. Most patients selected for this study were scanned using DECT in the emergency department, in the setting of abdominal pain and suspected biliary pathology. In addition, MRI was most often performed as follow-up on these patients because of suspected biliary abnormalities.

This study had some limitations. All imaging was performed at a single center using CT scanners from a single manufacturer. The majority of patients included in the study were scanned in the acute setting, either as emergency room or as inpatients. In this population, there might be a greater degree of underlying hepatic pathology. We did not have complete information about the causes of steatosis in all patients or the presence of other liver diseases, such as viral hepatitis or alcohol associated liver disease. Mineral deposition, such as iron and magnesium, and prior medication use, like amiodarone, may skew the results of DECT examinations. Retrospective study design may have introduced selection bias. We also did not consider additional clinical features of hepatic steatosis, such as liver function tests or other serum markers, in our evaluation.

Areas for future research include the impact of clinical and technical features on VNC liver attenuation and clinical outcomes in patients with such incidentally detected hepatic steatosis.

## Conclusion

VNC attenuation of the liver on contrast-enhanced DECT is highly specific for evaluation of hepatic steatosis and can be a promising tool for early, incidental detection of fatty liver disease.
